# Developing alcohol SBI policy and procedures with Alaska Public Health Nursing

**DOI:** 10.1186/1940-0640-10-S2-O32

**Published:** 2015-09-24

**Authors:** Rebecca Porter, D King, B Hanson, S Faulkner

**Affiliations:** 1 Center for Behavioral Health Research & Services, University of Alaska Anchorage, Anchorage, USA

## Background

The Institute of Medicine recommends changing organizational policy as a key element of evidence-based practice (EBP) implementation; however, policy change alone may be insufficient to change provider practice behavior[1,2]. Engaging key organizational stakeholders in the policy change process may foster buy-in, particularly if the policy is piloted and refined alongside the new practice. As part of a two-year initiative funded by CDC to implement routine alcohol screening and brief intervention (SBI) into primary care settings, the Alaska Section of Public Health Nursing (PHN) collaborated with the University of Alaska Anchorage to pilot alcohol SBI policy and procedures in three clinics prior to system-wide dissemination.

## Material and methods

Key PHN stakeholders and researchers formed a planning team to develop a draft alcohol SBI policy and nurse providers from pilot clinics were trained in the standardized procedures. Throughout the pilot, the university team maintained frequent contact with the clinics and monitored implementation through tracking data and contact notes. Findings were shared with the planning team regularly and the draft alcohol SBI policy and procedure was revised to balance fidelity to EBP with feasibility for PHN.

## Results

Screening rates, one measure of implementation success, increased throughout the pilot as barriers and challenges were identified and addressed, with corresponding updates to organizational policy and procedure. Nurses also indicated increased confidence and ability to conduct SBI as outlined in the policy. After fourteen revisions, the universal alcohol SBI policy and procedure was finalized and disseminated throughout Alaska's PHN system. Together, findings reveal that piloting and revising draft policy was an important implementation facilitator.

## Conclusions

Piloting and refining an organizational policy and procedure for alcohol SBI was effective for feasibility purposes and to implement the EBP with fidelity. Including nurses, leadership, and researchers throughout the implementation process fostered engagement in practice change.
